# Personality Styles of Dentists Practicing Hypnosis Confirm the Existence of the Homo Hypnoticus

**DOI:** 10.3389/fpsyg.2022.835200

**Published:** 2022-03-15

**Authors:** Thomas Gerhard Wolf, Elena Baumgärtner, Burkhard Peter

**Affiliations:** ^1^Department of Restorative, Preventive and Pediatric Dentistry, School of Dental Medicine, University of Bern, Bern, Switzerland; ^2^Department of Periodontology and Operative Dentistry, University Medical Center of the Johannes Gutenberg University of Mainz, Mainz, Germany; ^3^MEG-Stiftung München, Munich, Germany

**Keywords:** dentist, homo hypnoticus, homo hypnoticus dentalis, hypnosis, personality style, intuitive/schizotypal

## Abstract

Several publications with healthcare professionals, such as psychotherapists, have shown a significant difference in personality styles in practitioners using hypnosis compared to those not using hypnosis. To investigate differences in personality styles, dentists were contacted to participate in a personality-inventory [Personality Style and Disorder Inventory (PSDI)] online survey. Dentists using hypnosis (HYP DGZH) (*n* = 418) were compared to dentists not using hypnosis (NONHYP DENT) (*n* = 162). Results show that hypnosis-practicing dentists score significantly higher in the intuitive/schizotypal ST personality style (*p* < 0.001) compared to non-hypnosis-practicing dentists. Female dentists scored significantly higher in intuitive/schizotypal ST and unselfish/self-sacrificing SL in the HYP DGZH sample but only in unselfish/self-sacrificing SL in the NONHYP DENT sample. The percentage of women was similar in both samples (68.2%; 67.3%). Intuitive/schizotypal ST was found to be the predominant personality style of men who are interested in or use hypnosis, metaphorically so-called “homo hypnoticus.” Within the limitations of this cross-sectional non-interventional observational online-questionnaire study, results expand this notion to the so-called “homo hypnoticus dentalis.” However, further research on the subject is needed to investigate and confirm this personality type in other than the German-speaking DACH countries.

## Introduction

Since the first documented tooth extraction under hypnosis (Delatour, 1826), there is a considerable amount of literature on the diverse topics of dental hypnosis. After almost 200 years, the most recent report of a tooth extraction with hypnosis as the sole anesthesia was described by Cozzolino et al. (2020). Similar interventions have been reported repeatedly by Gheorghiu and Orleanu (1982) and Schmierer (1993). Due to the pioneering work of the latter (Schmierer, 1985, 2015), dental hypnosis received a growing reputation in the German-speaking DACH countries, such as Germany, Austria, and Switzerland, both in practical application (Mehrstedt and Wikström, 1997) and also increasingly in scientific research. For example, Wolf et al. (2016) presented a randomized, clinical-experimental crossover study for acute dental pain relief, and Halsband and Wolf (2016) reported on the functional changes in brain activity after hypnosis in patients with dental phobia. Also, outside the narrower circle of hypnosis journals, the topics of dental hypnosis were published (Wharton and Lewith, 1986; McKnight-Hanes et al., 1993; Verhoef and Sutherland, 1995; Dailey et al., 2001; Wannemueller et al., 2011). Apart from some earlier studies on the personality of dentists (e.g., McDaniel et al., 1988; Westerman et al., 1991; Needleman et al., 2011; Anil et al., 2017), a contemporary in-depth study of personality, similar to that of Peter et al. (2012) for hypnotherapists and Peter et al. (2018) for psychotherapists, has been lacking for hypnosis-dentists in particular, as well as for dentists in general.

Peter (2015), as well as Peter and Böbel (2020), presented studies that were indicative of a special personality profile of men interested in or using hypnosis in professional contexts. Peter (2015) coined the label “homo hypnoticus” for such people. The predominant personality style was found to be intuitive/schizotypal ST. The personality styles, such as unselfish/self-sacrificing SL, charming/histrionic HI, and optimistic/rhapsodic RH, also manifested repeatedly but not always reliably, e.g., in the study by Peter et al. (2014) or in a recent study with two samples of hypnotherapists and dentists who used hypnosis in their professional practice (Wolf and Peter, 2022). In contrast to the hypnotherapists, the hypnosis-dentists showed additionally a remarkably high level in the conscientious/compulsive ZW style. A major limitation of this latter study was the absence of a control sample of dentists not using hypnosis (NONHYP DENT). After many efforts, the data of an acceptable sample of dentists who do not use hypnosis could be collected only in the middle of 2021. This study aimed to compare the personality profiles of dentists using hypnosis (HYP DGZH) with that of NONHYP DENT to investigate if a personality profile of the so-called metaphorically “homo hypnoticus dentalis” may exist similar to the study by Peter and Böbel (2020) also metaphorically speaking. The homo hypnoticus (Peter and Böbel, 2020) is characterized with the predominant personality style intuitive/schizotypal, showed a tendency to rhapsodic/optimistic, and hints toward charming/histrionic.

The aim of this study was to investigate whether there were differences (1) between NONHYP DENT and HYP DGZH in the five interesting personality styles intuitive/schizotypal ST, unselfish/self-sacrificing SL, charming/histrionic HI, optimistic/rhapsodic RH, and conscientious/compulsive ZW (hypothesis 1) as well as (2) between the sexes of the two samples. Furthermore, we were interested in personality style differences (hypothesis 2) that exist (3) between low, medium, and high hypnotizables (hypothesis 3).

## Materials and Methods

### Study Samples

The HYP DGZH sample: As a sample of HYP DGZH in the professional area, approximately 1,348 members of the German Society of Dental Hypnosis [Deutsche Gesellschaft für Zahnärztliche Hypnose (DGZH), Stuttgart, Germany] were contacted. In addition, there were calls for participation in the survey during the congresses of the German and Austrian Dental Hypnosis Society (DGZH, ÖGZH) and also the Swiss Medical Society for Hypnosis (SMSH). A total of 418 subjects responded, 285 women (68.2%) and 133 men (31.8%) between 20 and 75 years of age (mean = 53.27; *SD* = 10.3). This hypnosis using a dentist sample (HYP DGZH) is identical to the sample of the 418 DGZH members examined in the study by Wolf and Peter (2022).

The NONHYP DENT sample: For the sample of dentists who were not practicing hypnosis, approximately 1,450 members of the universities of Bonn (Germany), Tübingen (Germany), Bern (Switzerland), Krems (Austria), and Vienna (Austria), as well as military dentists (Germany), were contacted. A total of 162 subjects responded, 109 women (67.3%) and 53 men (32.7%) between 21 and 69 years of age (mean = 37.99; *SD* = 10.8).

The study was designed as a cross-sectional non-interventional observational online-questionnaire study among dentists. All participants received an email that explained the study’s goals and asked for their participation in an investigation of personality styles *via* the Internet using the software SoSci Survey (SoSci Survey GmbH, München, Germany). No randomization or other group allocation was performed, and every participant was provided with written informed consent for research purposes. The data of the HYP DGZH sample were collected between August 1, 2017 and September 30, 2018, as well as between February 1, 2020 and June 30, 2020, and the data of the NONHYP DENT sample were collected between April 1, 2020 and April 30, 2021. The participation was entirely voluntary, and no advantage, financial or otherwise, was associated with participation. All procedures were performed in accordance with the 1964 Declaration of Helsinki and its subsequent amendments and the ethical standards of the local research commission. For this type of study, from the local ethics committee, no further formal approval was required. In accordance with the Swiss Human Research Act [810.30 Federal Law on Research Involving Human Subjects, Human Research Act (HRA)], the data from study participants were used under irreversibly anonymized conditions; all participants were adults.

### Measure

Similarly in the studies by Peter and Böbel (2020) and Wolf and Peter (2022), the short form of the Personality Style and Disorder Inventory (PSDI-S) by Kuhl and Kazén (2009) was used to evaluate the personality styles of the study participants. The PSDI is a questionnaire to self-report manifestations of 14 personality styles. The PSDI-S is standardized and provides the researcher with objective procedures and analyses. It shows reliability (Cronbach’s α = 0.64–0.79). The validation of this questionnaire has been established in several studies. The subjects answered 64 items on a 4-point Likert scale (from 1 = “strongly agree” to 4 = “strongly disagree”). The raw values were converted into *t*-values. The t-levels of 40–60 (one SD above/below the average mean of 50) describe personality styles, while those beyond this range band of 40–60 highlight the probability of a personality disorder. Of the 14 personality styles, only four interesting personality styles of the “homo hypnoticus,” namely, ST, SL, HI, and RH, and the ZW style for statistical calculations were analyzed. A short description of these five personality styles is found in Table 1. A detailed presentation of the PSDI used in several studies is given in the literature (Peter et al., 2018; Peter and Wolf, 2021).

**TABLE 1:** The 5 (of 14) scales of the Personality Styles and Disorders Inventory (Kuhl and Kazén, 2009) relevant to this study.

PSDI-scale*^a^*	Example
Intuitive/**schizotypal** ST	“There are supernatural forces”
Unselfish/self-sacrificing SL	“I am more concerned with other people’s worries than my own needs”
Charming/**histrionic** HI	“My good moods are very contagious to others”
Optimistic/rhapsodic RH	“I am an invincible optimist”
Conscientious/**compulsive** ZW	“Consistency and firm principles define my life”

*^a^DSM-5 or ICD-10 equivalents are indicated in bold.*

### Data Analysis

The results from the PSDI were analyzed using the software package IBM SPSS, version 27 (IBM, Armonk, NY, United States). The data were transferred from the questionnaire software SoSci Survey (SoSci Survey GmbH, München, Germany) directly in the data format for SPSS, respecting the currently applicable General Data Protection Regulation of the European Union (last accessed: January 12, 2022).^1^ The *t*-tests or one-way ANOVAs were used despite none of the PSDI scales showed normal distribution. Because, especially in large samples, both *t*-test and ANOVA are deemed to be quite robust against violations to the assumption of normality, we decided against the use of non-parametric tests, thus having more power detecting abnormalities and being able to calculate CIs for better assessment of the extent of these deviations. For evaluating the homogeneity of variances, Levene’s tests were used. Bonferroni tests of SPSS version 27 already adjusting for Type 1 Error were used for the *post hoc* analysis.

## Results

**FIGURE 1:**
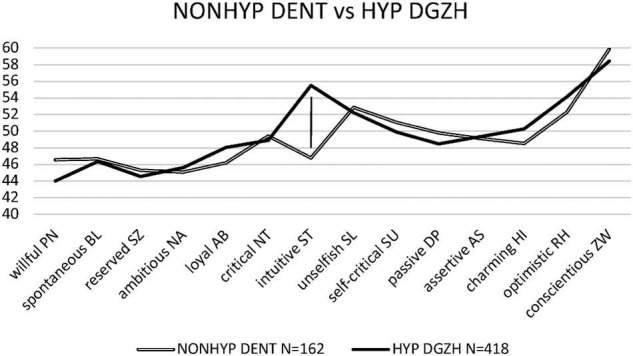
Personality profiles of dentists not using hypnosis (NONHYP DENT) and dentists using hypnosis (HYP DGZH).

For the difference between HYP DGZH and NONHYP DENT samples, the unpaired *t*-test revealed (with unequal variances) significant results only in the personality style intuitive/schizotypal ST [*t*(405) = 11.10, *p* < 0.001, *d* = 0.89, CI_0.95_ = 0.70/1.08] (Figure 1). After Bonferroni correction, the level of significance was missed in the unselfish/self-sacrificing SL style (*p* = 0.51), the charming/histrionic HI style (*p* = 0.047), the optimistic/rhapsodic RH style (*p* = 0.028), and the conscientious/compulsive ZW style (*p* = 0.043).

**FIGURE 2:**
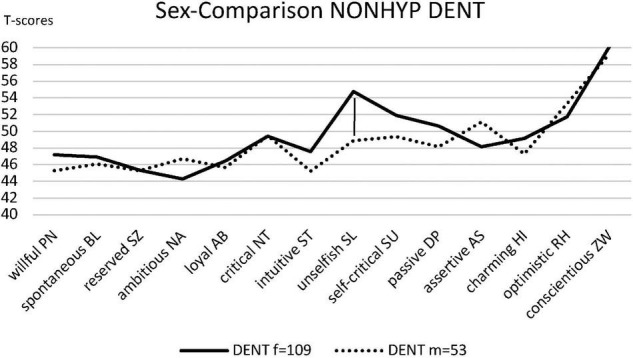
Personality profiles of the sexes of NONHYP DENT.

**FIGURE 3:**
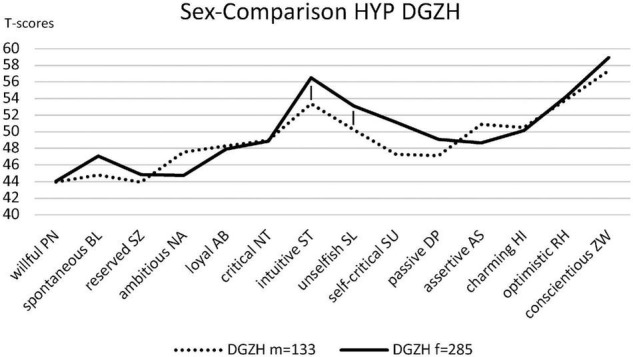
Personality profiles of the sexes of HYP DGZH.

For explorative reasons, the personality styles between women and men were tested within the respective groups. The personality styles of the NONHYP DENT sample (*n* = 162) consisting of 109 female and 53 male participants were compared in all fourteen styles. The analysis revealed that the female group scored higher in intuitive/schizotypal ST, unselfish/self-sacrificing SL, self-critical/avoidant SU, and passive/depressive DP traits compared to their male counterparts. However, after Bonferroni correction, only the difference in unselfish/self-sacrificing SL reaches statistical significance [*t*(123) = 3.78, *p* < 0.001, *d* = 0.59, CI_0.95_ = 0.26/0.93] (Figure 2). The sample of HYP DGZH (*n* = 418) consisted of 285 female and 133 male participants and showed significant differences only for the personality styles ST [*t*(416) = 2.86, *p* = 0.004, *d* = 0.30, CI_0.95_ = 0.03/0.57] and SL [*t*(304) = 2.88, *p* = 0.004, *d* = 0.28, CI_0.95_ = 0.01/0.56], not for HI, RH, and ZW (Figure 3).

**FIGURE 4:**
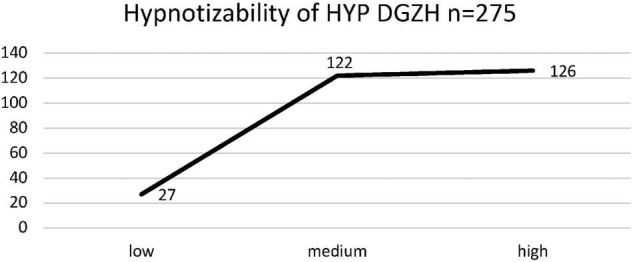
Number (*n*) of participants of HYP DGZH (sample) who knew about their level of hypnotizability (low, medium, and high).

**FIGURE 5:**
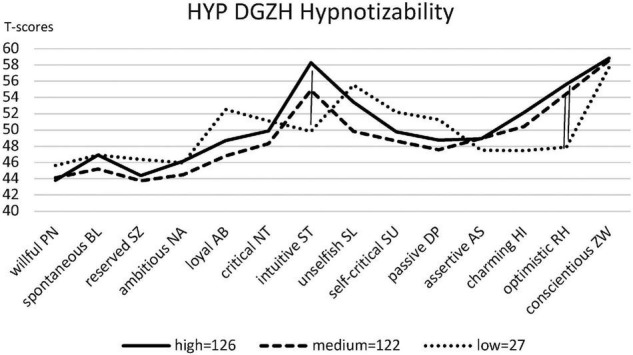
Personality profiles of the levels of hypnotizability of HYP DGZH (norm *T* = 50).

Of those 389 HYP DGZH study participants who stated that they used hypnosis in their work, 10 had attended only one or two seminars (16 h each), 76 attended more than two, and 303 attended more than 10 training seminars. Of these 303 who had attended more than 10 training seminars, 275 knew about their hypnotizability because they had been tested in the past. Notably, 126 were highly hypnotizable, 122 were medium hypnotizable, and 27 were low hypnotizable. Thus, for the hypnotizability of the HYP DGZH sample, there is no normal but an almost linear distribution with more high than medium and only a few low hypnotizables (Figure 4). The results of the ANOVA (equal variances) revealed that the hypnotizability levels of the HYP DGZH sample differed significantly concerning the intuitive/schizotypal ST style [*F*(5, 394) = 4.68, *p* < 0.001, η^2^ = 0.056, CI_0.95_ = 0.01/0.10], the unselfish/self-sacrificing SL style [*F*(5, 394) = 2.40, *p* = 0.036, η^2^ = 0.030, CI_0.95_ = 0.00/0.06], the charming/histrionic HI style [*F*(5, 394) = 3.24, *p* = 0.007, η^2^ = 0.039, CI_0.95_ = 0.00/0.07], and the optimistic/rhapsodic RH style [*F*(5, 394) = 4.07, *p* = 0.001, η^2^ = 0.049, CI_0.95_ = 0.01/0.09]. The Bonferroni-corrected post hoc analysis revealed significant differences for the intuitive ST style between the high and low hypnotizables (8.42, *p* = 0.002, CI_0.95_ = 2.00/14.85), for the optimistic RH style between the high and low hypnotizables (7.73, *p* = 0.001, CI_0.95_ = 2.10/13.37), and between the medium and low hypnotizables (6.58, *p* = 0.010, CI_0.95_ = 0.93/12.23) (Figure 5).

## Discussion

This study aimed to investigate the personality profiles of HYP DGZH in their professional environment to relieve patients’ anxiety, make phobics treatable, or even reduce acute or chronic pain, and compare those profiles to the ones of NONHYP DENT. The general result confirms the previous findings of the intuitive/schizotypal ST personality style being the predominant signature of the “homo hypnoticus,” i.e., people interested in hypnosis (Peter and Böbel, 2020; Figure 1). Because there were no significant differences in the other three styles unselfish/self-sacrificing SL, charming/histrionic HI, and optimistic/rhapsodic RH between the HYP DGZH and NONHYP DENT samples, these styles must probably be regarded not as essential but as accidental styles of the “homo hypnoticus.” This statement is reinforced by the astonishing explanation for this non-difference in this study: The values, specifically in unselfish/self-sacrificing SL and optimistic/rhapsodic RH, of the NONHYP DENT sample, were as high as those of the HYP DGZH sample, both of them more than two *t*-values higher than the norm mean of 50. Earlier in the study by Peter and Böbel (2020), charming/histrionic HI was not entirely characteristic for the “homo hypnoticus” because mainly caused by female participants. In this study, charming/histrionic HI values were equal in both samples and without difference from the norm mean of 50, thus most likely ruling out charming/histrionic HI as a characteristic of the “homo hypnoticus.”

There were three significant differences in personality styles when comparing female to male dentists (Figures 2, 3). Namely, unselfish/self-sacrificing SL scores were significantly higher in women in both samples, and the intuitive/schizotypal ST scores were significantly higher in women only in the HYP DGZH sample. Since gender distribution was very similar in both samples, one can conclude that women and men contributed to the unselfish/self-sacrificing SL style in both samples equally. Thus, in this study, unselfish/self-sacrificing SL is not a style that differs HYP DGZH from NONHYP DENT. This is different for the intuitive/schizotypal ST style where only in the HYP DGZH sample, women obviously contribute stronger than men. As they make up more than two-thirds of the test subjects, overall, we cannot decide whether this is just a product of statistical power or a genuine result such that the “homo hypnoticus” shows up mainly in women, as already suspected by Peter and Böbel (2020). A comparison of the demographic factors in HYP DGZH and NONHYP DENT samples showed that the HYP DGZH sample dentists were significantly older than NONHYP DENT dentists, which was most likely due to the different recruiting methods. Since personality styles are largely stable over the entire lifetime in adults (Leon et al., 1979), it is unlikely that this difference influenced the study results. Summarizing the data of this study and previous studies, we can confirm that intuitive/schizotypal ST is quite obviously the signature of the “homo hypnoticus,” i.e., people who are interested in hypnosis (Peter, 2015; Peter and Böbel, 2020).

By previous data, we have indications that this intuitive/schizotypal ST signature not only applies to those interested in hypnosis but also correlates with hypnotizability, in the study by Peter et al. (2014), however, only among the insecurely attached. Therefore, we asked the HYP DGZH dentists at the end of the questionnaire whether they suspected how well (low, medium, and high) they were hypnotizable or even knew this objectively because they had taken a hypnotizability test in the past. The result confirmed our assumptions regarding the styles intuitive/schizotypal ST and optimistic/rhapsodic RH: the highly hypnotizable subjects are significantly more intuitive/schizotypal ST and optimistic/rhapsodic RH than the low hypnotizable subjects; regarding optimistic/rhapsodic RH, moreover, the medium hypnotizable subjects also have higher values than the low hypnotizable subjects (Figure 5). Another result is interesting in this context: the normal distribution of hypnotizability, quite often reported in the literature, is not evident among our hypnosis-practicing dentists (HYP DGZH). On the contrary, only very few were low hypnotizable, but most were medium and high hypnotizable (Figure 4). This suggests that the normalization data of the hypnotizability scales may not be representative of the general population, as already claimed by Peter and Roberts (2022).

The intuitive/schizotypal ST style has been described as part of a continuum from the healthy population to extreme characteristics of schizophrenia with unusual perceptions and beliefs as one of its essential factors (Bentall et al., 1989; Claridge et al., 1996; Mohr and Claridge, 2015). Some authors have related hypnotizability to the “positive” aspects of schizotypy (Gruzelier, 1996; Jamieson and Gruzelier, 2001). Connors et al. (2014) even discussed due to their own data hypnotic analogs in delusion proneness and schizotypy. However, the very benign endpoint on the schizotypy scale is intuition, which is in the opinion of the authors the prerequisite for a good, rapport-based application of hypnosis by both psychotherapists and dentists. In this respect, the predominance of the intuitive/schizotypal ST personality style among these representatives of the “homo hypnoticus” is not surprising. However, Peter and Böbel (2020) have already pointed out that it is possibly this very intuitive/schizotypal ST style that makes natural scientists look skeptically at hypnosis and its practitioners when they equate it with esotericism.

### Limitations

We deliberately selected the two samples to find differences: The HYP DGZH group was contacted with clear references to hypnosis, whereas any reference to hypnosis was avoided in the NONHYP DENT group. However, we could not avoid the self-selection bias due to the principally voluntary participation in the study. This may have contributed, among other things, to the other major limitation of our study, i.e., the very small sample of the control group of the NONHYP DENT sample. Although there were many dentists contacted over 1 year, the willingness to participate in the online self-questionnaire was scarce. Reasons could be that dentists are not as accustomed as psychologists to spend time as test subjects. The time required to complete the questionnaire is approximately 20 min, which is rather in the upper third compared to common questionnaire studies in dentistry. This could mean that the 162 NONHYP dentists in our study have high unselfish/self-sacrificing SL scores for this very reason and therefore may not be representative of dentists in general. However, it is precisely for this reason that their significantly low intuitive/schizotypal ST values contribute to the difference to the significantly high intuitive/schizotypal ST values of their HYP DGZH dentists. A further limitation is that the selected target samples consisted only of dentists from the German-speaking DACH countries, such as Germany, Austria, and Switzerland. Other populations from other regions of the world, especially of other continents, should be investigated for this specific personality style both in dentists and in hypnosis using healthcare professionals in general.

## Conclusion

Several studies have shown that healthcare professionals of different disciplines, such as psychotherapists, using hypnosis within the context of their professional area show significant differences in certain personality styles compared to their peers not practicing hypnosis. This led to the formulation of the “homo hypnoticus” hypothesis (Peter et al., 2014; Peter, 2015; Peter and Böbel, 2020). The study presented compared HYP DGZH and NONHYP DENT. The results confirmed the predominance of the intuitive/schizotypal ST personality style for the HYP DGZH sample in contrast to their NONHYP DENT counterparts. Within the limitations of this cross-sectional non-interventional observational online-questionnaire study, the results underline the existence of the so-called “homo hypnoticus” also among dentists, the so-called “homo hypnoticus dentalis.” However, further research on the subject is needed to further investigate and confirm this personality style by other studies.

## Data Availability Statement

The raw data analyzed in this study are subject to the following licenses/restrictions: General European Data Protection Regulation (GDPR) of 25 May 2018. Requests to access these datasets should be directed to PB, burkhard-peter@t-online.de.

## Ethics Statement

Ethical review and approval was not required for the study on human participants in accordance with the local legislation and institutional requirements. The patients/participants provided their written informed consent to participate in this study.

## Author Contributions

BP and TW designed and planned the study. EB and TW were in charge of the experiment. BP was in charge of the statistical conduction of the investigation. EB prepared the first draft of the manuscript. TW and BP revised the first draft of the manuscript. All authors read and approved the final manuscript.

## Conflict of Interest

The authors declare that the research was conducted in the absence of any commercial or financial relationships that could be construed as a potential conflict of interest.

## Publisher’s Note

All claims expressed in this article are solely those of the authors and do not necessarily represent those of their affiliated organizations, or those of the publisher, the editors and the reviewers. Any product that may be evaluated in this article, or claim that may be made by its manufacturer, is not guaranteed or endorsed by the publisher.

## Footnotes

1
https://gdpr.eu
